# Nanoscale Investigation of Elasticity Changes and Augmented Rigidity of Block Copolymer Micelles Induced by Reversible Core-Cross-Linking

**DOI:** 10.1021/acsami.5c04826

**Published:** 2025-04-23

**Authors:** Xinyue Wang, Andreas Stihl, Christiane Höppener, Jürgen Vitz, Felix H. Schacher, Volker Deckert

**Affiliations:** † Institute of Physical Chemistry and Abbe Center of Photonics, Friedrich-Schiller University, D-07743 Jena, Germany; ‡ Leibniz Institute of Photonic Technology, D-07745 Jena, Germany; § Institute of Organic Chemistry and Macromolecular Chemistry, Friedrich-Schiller University, D-07743 Jena, Germany; ∥ Jena Center for Soft Matter (JCSM), Friedrich-Schiller University, Philosophenweg 7, D-07743 Jena, Germany

**Keywords:** atomic force microscopy, nanoindentation, block copolymer micelles, reversible cross-linking, nanomechanical properties, mechanical stability

## Abstract

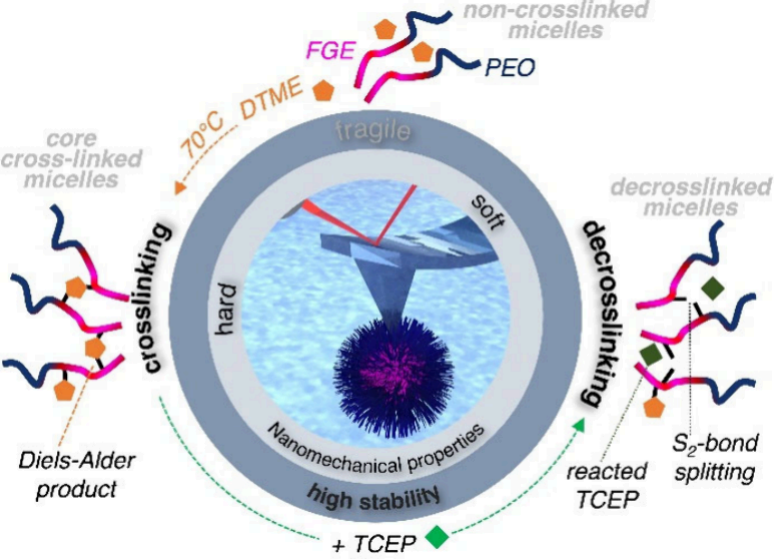

Drug-delivery systems have attracted considerable attention due to their potential to increase the bioavailability of certain drugs and mitigate side effects by enabling targeted drug release. Reversibly core-cross-linked block copolymer micelles providing a hydrophilic and potentially nonimmunogenic shell and a hydrophobic core suitable for the uptake of hydrophobic drugs are frequently considered because of their high stability against environmental changes and dilution. Ultimately, triggering core-de-cross-linking enables the implementation of strategies for targeted drug release, which requests insights into the impact of varying nanomechanical properties on the stability of individual micelles. Here, atomic force microscopy nanoindentation in aqueous media is applied to intact α-allyl-PEG_80_-*b*-P(*t*BGE_52_-*co*-FGE_12_) micelles to quantify changes in their nanomechanical properties induced by dithiobismaleimidoethane (DTME)-mediated Diels–Alder cross-linking of furfuryl moieties and sequential de-cross-linking by reduction of its disulfide bond by tris(2-carboxyethyl)phosphine. As a result of crosslinking by DTME, the apparent Young’s modulus of the micelles roughly doubles to 1.18 GPa. Changes to the Young’s modulus can be largely reversed by de-cross-linking. Cross-linked and de-cross-linked micelles maintain their structural integrity even in diluted aqueous media below the critical micelle concentration, in contrast to the micelles prior to crosslinking. Understanding the structure–property relationships associated with the observed augmented mechanical stability in native environments is crucial for improving the efficiency of drug encapsulation and introducing refined temporal and spatially controlled drug-release mechanisms.

## 1 Introduction

The encapsulation of hydrophobic drugs in block copolymer micelles acting as drug-delivery systems (DDSs) is motivated by multiple factors, e.g., by increased bioavailability or by introducing targeted release strategies to reduce harmful side effects.^1−3^ Additionally, drug-loaded nanoparticles exhibit an enhanced permeability and retention effect, allowing the passive targeting of tumors.^4^ Amphiphilic block copolymers including hydrophilic and hydrophobic blocks self-assemble to form various structures depending on different parameters,^5^ such as encompassing hydrophobic interaction,^6^ hydrogen bonding,^7^ and electrostatic interactions.^8^ In particular, the formation of nanosized core–shell block copolymer micelles in selective solvents is very interesting, in view of drug-delivery applications. In the fundamental polymeric micellar architecture, the hydrophobic part of the amphiphiles congregate in a hydrophobic core, shielded from the aqueous environment, while the hydrophilic segments interface with the surrounding water.^9^ This arrangement imparts stability to the micelle by minimizing the exposure of hydrophobic components to an aqueous environment. This configuration is advantageous for encapsulating hydrophobic compounds within the micellar core, providing a stable and solubilized environment facilitating high shell-medium compatibility.

In the quest to improve the stability of micelles, a range of hydrophilic and flexible polymers, including poly(ethylene glycol) (PEG),^3^ poly(vinyl alcohol),^10^ and polysaccharides,^11^ act as key components to form the micellar shell. A meticulous design of the chemical composition within the core segments is crucial to ensuring alignment with the specific characteristics of the target drug molecules.^12^ Therefore, the formation of micelles and the encapsulation of payloads hinge on the fundamental separation of the core from the aqueous environment.

PEG is a common choice for the hydrophilic block in block copolymer micelles used for drug encapsulation due to its solubility in water, its nonimmunogenicity and its ability to prevent unwanted protein or cell adhesion when used as an outer layer for surfaces or particles.^1,13^ However, PEG-based block copolymer micelles face several challenges when used as drug carriers. First, micelles can dissociate and release their encapsulated cargo prematurely as a result of dilution below the critical micelle concentration (CMC),^14^ changes in the pH or temperature,^15^ or the presence of substances, which interact with the polymer chains.^16^ Second, the presence of the hydrophobic block can increase the immunogenicity of the PEG-based micelles, triggering the release of anti-PEG IgM antibodies, leading to the accelerated blood clearance phenomenon.^3^ This has been observed in animal trials, with micelles exceeding 50 nm showing a greater propensity for causing this effect.^17,18^

Resisting dissociation encountered under highly diluted conditions is a crucial factor in the development of self-assembled systems for drug delivery. Consequently, for an effective usage of PEGylated block copolymer micelles as drug carriers, their stability as well as effective masking of the interface between the hydrophilic and hydrophobic blocks must be maintained throughout the administration process; i.e., the micelles must retain their integrity. One way to achieve this is to use a material with a high *T*_g_ for the hydrophobic core,^19^ leading to a frozen-in micellar structure. However, this approach has been associated with a limited loading capacity in the core because the frozen-in structure is unable to rearrange to encapsulate a payload.^20^ Cross-linking, particularly core-cross-linking of block copolymer micelles to reinforce their mechanical stability, is considered to be an effective approach toward the preservation of a desired morphology or the chemical integrity even in nonselective environments.^5^ Prospectively, reversible core-cross-linked (CCL) strategies can be exploited to establish controlled-drug-release mechanisms by initiating de-cross-linking reactions after cellular uptake. Recently, reversible CCL micelles have become a topic of interest.^15,21,22^ Introducing destabilization or disassembly by the application of certain stimuli, such as changes in the pH, temperature, or application of a de-cross-linking agent, may induce drug release. In the biological context, reductive cleavage of disulfide bonds is of particular importance because this binding motif is present in a number of proteins. Targeted release can reduce the potential side effects of encapsulated drugs.^15,23^

Within this context, the examination of micelles composed of block copolymers, incorporating PEG as the hydrophilic block and polymers with furfuryl pendant groups as the hydrophobic block, has been a subject of in-depth investigation.^22−25^ These polymers are easily synthesized using a PEG-containing macroinitiator through either anionic ring-opening (co)polymerization (AROP) of furfuryl-containing glycidyl ethers (FGEs) or controlled radical (co)polymerization of a suitable furfuryl-containing monomer.^22,25^ Importantly, these can then be reversibly cross-linked under mild conditions via a Diels–Alder (DA) reaction with disulfide- or diselenide-bridged bismaleimides by heating to 60–70 °C and sequential de-cross-linking by reduction of the disulfide or diselenide bridge.^23−25^ Comprehending the intricacies of the cross-linking process and discerning the effects of reversible cross-linking on the functionality of these micelles as DDSs is crucial for advancing their clinical applications and require knowledge on the nanoscale structure–property relationships.

**Figure 1:**
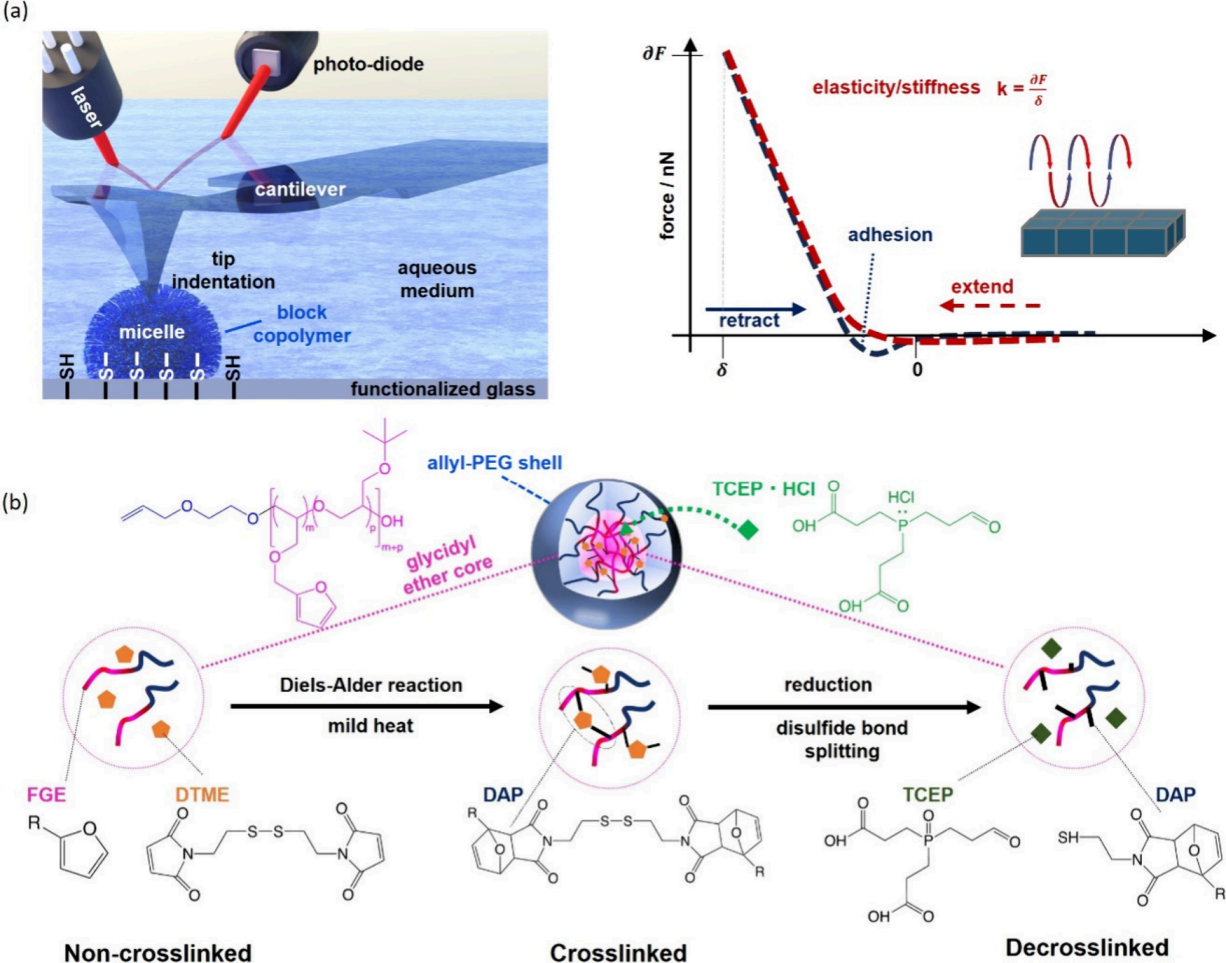
(a) Schematic illustration of liquid nanoindentation AFM investigations of immobilized, intact α-allyl-PEG_80_-*b*-P(*t*BGE_52_-*co*-FGE_12_) micelles (left) for quantification of their nanomechanical properties from force–distance curves (right). Micelles are immobilized to mercaptosilane-functionalized glass substrates with thiol–ene chemistry. (b) Schematic outline of the micellar core–shell structure and the chemical reaction pathways from non-cross-linked micelles to CCL micelles by a DA reaction and de-cross-linked micelles by disulfide bond splitting (left to right). PEG = poly(ethylene glycol), FGE = furfuryl glycidyl ether, DTME = dithiobismaleimidoethane (cross-linker), DAP = Diels–Alder product, and TCEP = tris(2-carboxyethyl)phosphine hydrochloride (de-cross-linker).

Advanced analytical techniques, particularly atomic force microscopy (AFM) imaging and AFM nanoindentation, are valuable tools providing information on nanoscale morphology, e.g., the size, shape, and structural homogeneity, and can pair these with the nanoscale elasticity and adhesion. AFM nanoindentation is a specialized imaging technique within the realm of AFM. By recording force–distance curves at each pixel of an AFM topography image, this method enables the quantification of material properties (Figure 1a), thereby providing nanoscale insights into surface characteristics and molecular interactions.^26−30^ AFM nanoindentation operates on the principle of measuring the forces between a sharp tip and a sample surface as a function of their relative separation distance (Figure 1a). When the tip is subjected to a controlled force and the resultant deflection of the cantilever is monitored, the interaction forces are systematically quantified. This process yields crucial information, including but not limited to determination of Young’s modulus, a key parameter in characterizing the material elasticity. Changes in Young’s modulus are analyzed based on the correlation of tip indentation and applied force in the elastic regime. Low loading rates and small indentation depths enable the study of the nondestructively local mechanical properties of soft and fragile nanoscale materials like cells, tissues, hydrogels, and others^31−35^ and visualization of the distribution of carbon nanotubes and nature rubber regions in nanocomposites.^36^ AFM nanoindentation applied to block copolymer micelles determined the influence of the crystallinity of the micellar corona on the bending modulus,^37^ provided insights into the nanomechanical properties of single Casein micelles upon variations of the physical–chemical conditions,^38^ and revealed parameters affecting the efficiency of chemical reactions in the confined core region,^39^ showing its capability to extract nanoscale information on individual micelles. Furthermore, AFM nanoindentation studies of multiple self-assembled biopolymer fibers allowed the reveal of their morphological and mechanical heterogeneity and enabled the identification of cross-linking-induced structural changes.^40−45^ The reliability of AFM indentation experiments and the ability to extract local mechanical property gradients have been proven in numerous studies of different materials, transforming AFM nanoindentation into a powerful tool even for nanoscale analysis of heterogeneous polymers and nanostructures thereof.^46^ Therefore, AFM nanoindentation investigations enable the quantitative study of the (de-)cross-linking process at the individual micelle level in terms of elasticity changes. Although the well-known bottom effect artifact (BEA)^35,47^ induced by the stiffness of the supporting substrate usually results in an overestimation of the real Young’s modulus values, the relative changes between cross-linked and de-cross-linked micellar states considered here provide reliable trends, as discussed below in the Results and Discussion. Because the morphological integrity and chemical reaction mechanisms associated with the (de-)cross-linking processes are governed by environmental interactions with the PEG shell and the PEG–glycidyl ether interface, investigations of intact micelles are more reasonable for the sequential improvement of their structure–property relationships and functionality as potential drug carriers. Therefore, the present study focuses on the AFM and nanoindentation investigation of intact micelles in an aqueous environment, demanding sufficient immobilization of the micelles on a substrate.

Utilizing an allyl-PEG as the macroinitiator for synthesis of the block copolymer enables the immobilization of micelles on thiol-decorated surfaces by means of thiol–ene click reactions. To establish reversible crosslinking under mild conditions, dithiobismaleimidoethane (DTME) is used, which fosters cross-linking by means of a DA reaction and enables de-cross-linking by application of the reducing agent tris(2-carboxyethyl)phosphine hydrochloride (TCEP; Figure 1b). AFM nanoindentation tracks changes in the morphology, size, homogeneity, and mechanical properties of intact micelles containing freely diffusing DTME before (from now on denoted as non-cross-linked micelles) and after initiation of the crosslinking and de-crosslinking processes in an aqueous environment. The morphology, size, and homogeneity of non-crosslinked, crosslinked, and de-crosslinked micelles are compared with independently obtained results from standard characterization tools, e.g., dynamic light scattering and cryogenic transmission electron microscopy (cryo-TEM). Furthermore, supporting Information related to the chemical composition and crosslinking efficiency are approached by surface-enhanced Raman scattering (SERS) and high-resolution magic-angle-spinning (HR-MAS) NMR spectroscopy. This enables the direct study of micelle changes in response to chemical stimuli, allowing predictions concerning the stability of the micelles and, prospectively, their potential ability for releasing encapsulated molecules.

## 2 Materials and Methods

### 2.1 Preparation of Cross-Linked Micelles (General Procedure)

Tetrahydrofuran (THF) was purchased from VWR and purified under reduced pressure. Allyl-PEG_80_-*b*-P(*t*BGE_52_-*co*-FGE_12_) was synthesized according to the procedures described in the Supporting Information and dissolved in THF at a concentration of 2 mg/mL, along with DTME (1 equiv vs FGE groups). An equal volume of micropure water was added over 2 h using a syringe pump, and the mixture was stirred while open to the air until THF had evaporated (solvent switch). To cross-link, the solution was placed into a sealed vial and heated to 70 °C overnight.

For use in ^1^H HR-MAS NMR, this procedure was conducted with varying amounts of DTME on a scale of 30 mg of polymer. After dialysis against water (MWCO = 1 kDa) to remove unencapsulated DTME, the resulting solution was freeze-dried after a sample had been taken and swelled using CDCl_3_.

### 2.2 Nuclear Magnetic Resonance (NMR)

^1^H NMR spectra were measured on a Bruker AC 300 MHz instrument using deuterated solvents. HR-MAS NMR measurements were conducted using a Bruker Avance III HD 500 MHz spectrometer with a rotational frequency of 4000 Hz and with samples being swelled using CDCl_3_. Spectra were referenced to the residual peak of the solvent, phase-corrected using Mestrenova’s “Regions” algorithm, and automatically baseline-corrected via Bernstein polynomials. Degrees of cross-linking were determined using the molar ratios of the Diels–Alder product (DAP), unreacted furfuryl units, and unreacted maleimide units, following the described procedure in ref (22) (see also Supporting Note 2.1).

### 2.3 Size-Exclusion Chromatography (SEC)

For the polymer α-allyl-ω-hydroxy-PEG, SEC was performed using a SEC Agilent 1200 series system, a controller with a G1310A pump, a G7162A refractive index detector, and a PSS GRAM guard/30/1000 Å column (10 μm particle size) tempered at 40 °C. The eluent was a mixture of dimethylacetamide with lithium chloride (0.21 wt %) pumped at a flow rate of 1 mL/min. The SEC system was calibrated with PEG/PEO standards from Polymer Standards Service GmbH (PSS, Mainz, Germany). MALDI-TOF mass spectra were obtained utilizing an Ultraflex III TOF/TOF mass spectrometer (Bruker Daltonics) used in a reflector as well as linear mode with dithranol or 2,5-dihydroxybenzoic acid (DHB) as the sample matrix. The instrument was calibrated prior to each measurement with external poly(methyl methacrylate) standards in the range of 2500–10000 g/mol (PSS, Mainz, Germany).

For the polymer α-allyl-PEG-*b*-P(*t*BGE-*co*-FGE), SEC traces were conducted using an Agilent 1260 series system, equipped with a 1260 IsoPump (G1310B), a 1260 ALS (G1310B) autosampler, and three consecutive PSS SDV, 5 μm, 8 × 300 mm columns (100, 1000, and 100000 Å). THF was used as an eluent at a flow rate of 1 mL/min. The column oven was set to 30 °C, and signals were detected using a 1260 RID (G1315D) detector. The system was calibrated using PSS PEG/PEO (238–969000 g/mol) standards.

### 2.4 Dynamic Light Scattering (DLS)

DLS measurements were conducted at a scattering angle of 90° using a light scattering setup equipped with an ALV-SP125 goniometer, an ALV/LSE5004 multi-τ correlator, a fiber optical ALV/High QE APD avalanche photodiode with pseudocross correlation (ALV-Laservertriebsgesellschaft mbH, Langen, Germany), and a uniphase He/Ne laser (632.8 nm, Thorlabs Inc.). The temperature was controlled by a Huber Pilot One thermostat (Peter Huber Kältemaschinenbau AG, Offenburg, Germany) and kept at 25 °C. Correlation was recorded over three runs of 30 s, with runs containing count rate spikes of more than 2 times the average count rate being discarded because such spikes are likely caused by contaminants. Number-weighted size distributions were obtained using the CONTIN algorithm, and the peaks were evaluated using *OriginPro 2023b*, via the QuickPeaks algorithm, assuming a normal distribution. Hydrodynamic radii were determined by using a minimum of three measurements. The purpose of the measurement was determination of the size of the intact micelles; therefore, in the case of a multimodal size distribution, the peak in the range consistent with the monomodal size distribution (*r*_H_ = 10–30 nm) was used for micelle size determination.

A micelle solution of 1 g/L was prepared at a scale of 16 mg of polymer with 1 equiv of DTME. After any evaporated water was refilled, the micelle solution was heated to 70 °C overnight in a microwave vial to cross-link the micelles. The resulting solution was filtered using a 0.45 μm Nylon-66 syringe filter and split into four equal portions. Overnight, these were incubated with varying amounts of TCEP (1, 2, and 4 equiv vs DTME, with one portion as the control), followed by sequential dialysis against water and methanol (MWCO = 1 kDa) and filtration by 0.45 μm Nylon-66 syringe filters. DLS measurements were performed before and after cross-linking and after dialysis of the de-cross-linked solutions with both water and methanol using samples filtered with 0.45 μm Nylon-66 syringe filters. This experiment was performed in triplicate.

### 2.5 Transmission Electron Microscopy (TEM)

TEM micrographs were recorded using a 200 kV FEI Tecnai G2 20 equipped with a 4K × 4K Eagle HS CCD and a 1K × 1K Olympus MegaView camera (Thermo Fisher, USA). Samples were prepared by the deposition of 10 μL of the sample solution onto Quantifoil carbon support films using a Cu-400 support mesh for dry TEM and a 3.5/1 Cu mesh for cryo-TEM. Cryo-TEM samples were blotted and frozen by plunging into freezing liquid ethane using a Vitrobot Mark IV before being stored under liquid nitrogen and transferred to the microscope using a Gatan transfer stage. Micrographs were evaluated using the software *ImageJ*.

### 2.6 AFM Imaging and AFM Nanoindentation

AFM and AFM nanoindentation measurements of DA-CCL as well as 24h-de-cross-linked micelles were performed by a Nanowizard 3 SPM system (Bruker-JPK, Germany). HA_NC-B cantilevers with a resonance frequency of 140 kHz and a spring constant of 3.5 N/m were used (typical values as provided by the manufacturer NT-MDT).

All glass substrates were cleaned with a mixture of H_2_O_2_ and HNO_3_ (volume ratio = 1:3) for 2 h, then rinsed with H_2_O, and dried with an argon flow. AFM sample characterization was carried out in ambient conditions as well as in liquid. For the ambient AFM measurements, the micelle solution was drop-coated onto a clean glass substrate. Immobilization of the micelles for the liquid AFM and nanoindentation measurements were done on (3-mercaptopropyl)trimethoxysilane-functionalized glass substrates. The glass was silanized by vapor deposition. This process involved heating at 75 °C for 1 h, followed by an overnight incubation at room temperature and sequential cleaning steps with toluene and deionized water. Micelle solutions of 10 μL were dropped on silane-functionalized glass, and an equivalent volume of a 10 mg/mL lithium phenyl-2,4,6-trimethylbenzoylphosphinate solution was added. The sample was then kept in a humidity chamber and exposed to UV-laser radiation (18 W) for 30 min. The immobilized samples were washed with H_2_O and fixed on the sample holder immediately without further drying. A total of 10 μL of a 1.5 mg/mL TCEP aqueous solution was dropped onto the immobilized micelle samples. After 24 h of incubation, de-cross-linking was terminated by removing excess TCEP and washing with H_2_O.

AFM images were postprocessed using the open-source SPM data processing software *Gwyddion* (version 2.61).^48^ Processing steps comprise plane and line flattening procedures and adjustment of the image contrast.

Nanoindentation measurements were conducted in QI mode with a maximum applied force of 1.5 nN, a *z* length of 75 nm, and a pixel time of 5.5 ms. For each sample, three 500 × 500 nm^2^ (each with 256 × 256 pixels) Young’s modulus maps of different positions were recorded. Maps of the relative Young’s modulus were extracted from the slope of the extended force–distance curves observed in the contact region by fitting each force distance curve with an implemented Hertz–Sneddon model^49^ provided by the JPK data processing software (version 6.1.158). The fitting of the data considers a conical tip shape with an opening half-angle of 15°. The corresponding values of Young’s moduli are plotted for each image pixel and represent apparent Young’s modulus values.

To estimate the Young’s moduli of the micelles, only areas with heights of >10 nm in topography were considered by applying a corresponding mask to the Young’s modulus image. The corresponding values of Young’s modulus of the masked regions were extracted from each image using the open-source SPM data processing software *Gwyddion* (version 2.61). These values were graphically represented as box plots using *OriginPro 2022* for statistical analysis. Statistical evaluation of the height and Young’s modulus changes used >58000 force distance curves for each sample.

## 3 Results and Discussion

### 3.1 Micellization and Core-Cross-Linking and De-Cross-Linking of α-Allyl-PEG-*b*-P(*t*BGE-*co*-FGE)

**Figure 2:**
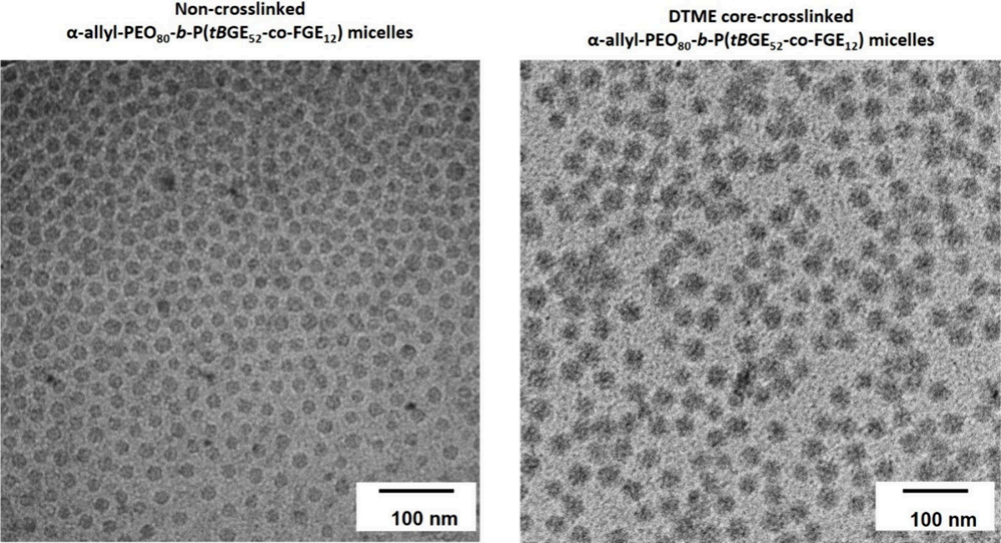
Cryo-TEM images of the non-cross-linked (containing unreacted DTME) (left) and DTME CCL α-allyl-PEG_80_-*b*-P(*t*BGE_52_-*co*-FGE_12_) micelles (right).

Briefly, α-allyl-PEG_80_-*b*-P(*t*BGE_52_-*co*-FGE_12_) (Figure 1b) was synthesized via chain extension by AROP (see Supporting Note 1.2) using α-allyl-ω-hydroxy-PEG_80_ as a macroinitiator (see Supporting Note 1.1). Micelles were produced via a solvent switch to water from a THF solution of the polymer and DTME as a cross-linker (polymer concentration 2 mg/mL). Cross-linking was achieved via a DA reaction between furfuryl and maleimide moieties by heating of the micelle solution to 70 °C overnight (Figure 1b). Cryo-TEM images of the non-cross-linked and CCL samples (Figure 2) reveal spherical micelles with a diameter of ∼40 nm and a homogeneous size distribution.

**Figure 3:**
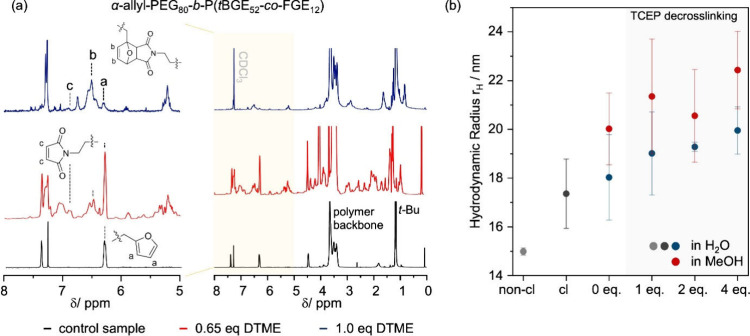
(a) ^1^H HR-MAS NMR measurement of DTME-cross-linked allyl-PEG_80_-*b*-P(*t*BGE_52_-*co*-FGE_12_) micelles with 0.65 equiv (red) and 1.0 equiv (blue) of DTME vs FGE groups. For comparison, an ^1^H HR-MAS NMR spectrum (black) of the polymer is shown. Magnified spectra (left) show the signals used to determine the molar ratios between the DAP and unreacted furfuryl and maleimide units. (b) Mean hydrodynamic radii *r*_H_ of micelle solutions of allyl-PEG_80_-*b*-P(*t*BGE_54_-*co*-FGE_14_) with 1 equiv of DTME before [non-cross-linked (noncl), light gray] and after cross-linking (cl, dark gray) after incubation of the latter with varying amounts of TCEP (1, 2, or 4 equiv vs DTME) and sequential dialysis against water (blue) and methanol (red). To exclude that *r*_H_ variations are imposed by the dialysis step, corresponding control samples without TCEP (0 equiv vs DTME) are shown additionally. Error bars correspond to the standard deviation (*n* = 3).

The degree of cross-linking of freeze-dried samples obtained from micelle solutions containing about 30 mg of polymer, with varying amounts of DTME was assessed via ^1^H HR-MAS NMR. (Figure 3a). Based on the results, optimal cross-linking occurred around 1 equiv of DTME vs FGE groups, with a maximum degree of cross-linking of 75–88% being observed (Table S1). This is in contrast to results described for similar polymers cross-linked with 1,1′-methylenedi-4,1-phenylenebismaleimide (BMA), where 0.65 equiv of BMA resulted in a degree of cross-linking of 60%.^22^ The same amount of DTME in the current case results in a degree of cross-linking of only 35–50%, while for 1.5 equiv of DTME, the degree of cross-linking could not be determined. It is likely that, compared to BMA, DTME has a lower affinity for the hydrophobic core of PEG-*b*-P(*t*BGE-*co*-FGE) micelles due to the missing phenyl groups, which enable π–π stacking with furfuryl groups in side chains, such that a higher concentration of DTME is required to achieve an equivalent accumulation of the cross-linker in the micellar core, while an excessive amount of cross-linker disrupts the micellar structure. This disruption is likely caused by an overall decreased cross-linking efficiency and increased monoaddition of the cross-linker, leading to an increased core volume and lower core stability. Nevertheless, the cross-linking efficiency can be precisely accessed by HR-MAS NMR via detection of the DAP, and de-cross-linking cannot be approached similarly due to splitting of the disulfide bond, which does not affect the DAP. Notably, DA-cross-linked micelles usually show good long-term stability. According to our general experience, these types of cross-linked micelles can remain intact over several months in selective solvents. Notably, no visible change in the DA-cross-linked micellar solutions was observed during storage in snapcap vials on the benchtop over several months.

Therefore, preliminary DLS investigations of micelles prior to initiation of the DA cross-linking process, as well as of DTME cross-linked and TCEP de-cross-linked micelles, were carried out in selective and nonselective solvents (Figures 3b and S1–S3). Such investigations provided an initial estimate of their size and insights into the impact of the applied chemical reactions on the mechanical stability. The hydrodynamic radius of the non-cross-linked micelles containing unreacted DTME was 15.0 ± 0.2 nm, coinciding well with the diameter observed in the cryo-TEM data. After DTME cross-linking, the hydrodynamic radius changed to 17.4 ± 1.4 nm. Transferring these cross-linked micelles into methanol as a nonselective solvent led to a swelling of the micelles (*r*_H_ = 20.0 ± 1.5 nm) compared to micelles kept in a selective environment. Despite swelling, the micelles seemed to retain their morphological integrity. No visual changes were observed in micelle solutions in either solvent during sample storage over several days in snapcap vials on the benchtop. Similarly, slight swelling occurred when aqueous micelle solutions were incubated with different amounts of TCEP (*r*_H_ = 19.0 ± 1.7 nm for 1 equiv of TCEP, 19.3 ± 0.2 nm for 2 equiv of TCEP, and 20.0 ± 1.0 nm for 4 equiv of TCEP). When these micelles were transferred to methanol, additional slight swelling occurred (*r*_H_ = 21.4 ± 2.4 nm for 1 equiv TCEP, 20.6 ± 1.9 nm for 2 equiv of TCEP, and 22.4 ± 1.6 nm for 4 equiv of TCEP). A closer inspection of the DLS measurements (Figure S3) revealed some instances with a bimodal distribution exhibiting an additional peak related to a hydrodynamic radius *r*_H_ of ∼10 nm, likely indicating partial disassembly of the de-cross-linked micelles in these conditions (see Supporting Note 2.2 for further details). While the hydrodynamic radii obtained for the de-cross-linked micelles in water and methanol were within the standard deviation for any given amount of TCEP, the values obtained for the micelles in methanol were consistently higher than those obtained for micelles in water, indicating swelling upon solvent exchange.

### 3.2 Morphology and Chemical Characterization of α-Allyl-PEG_80_-*b*-P(*t*BGE_52_-*co*-FGE_12_) Micelles

**Figure 4:**
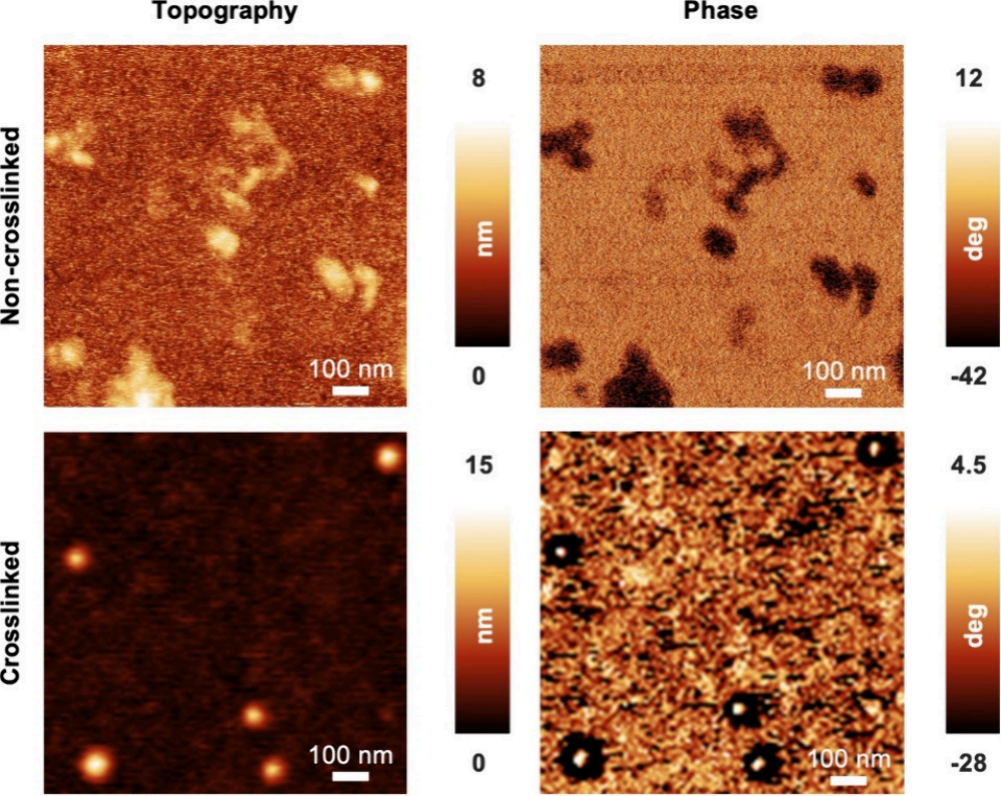
AFM topography and phase images of the non-cross-linked (containing unreacted DTME) (top) and DTME CCL α-allyl-PEG_80_-*b*-P(*t*BGE_52_-*co*-FGE_12_) micelles (bottom). AFM investigations were carried out under ambient conditions.

Although ^1^H HR-MAS NMR spectroscopy determines the cross-linking yield and DLS provides insights into the mechanical stability, these investigations cannot provide information on mechanical property changes related to the core-cross-linking and de-cross-linking processes. AFM investigations of dried samples of the non-cross-linked and DTME-cross-linked α-allyl-PEG_80_-*b*-P(*t*BGE_52_-*co*-FGE_12_) micelle samples show distinct morphological differences and varying material properties (Figure 4). After immobilization of the micelles and a sequential drying step, the non-cross-linked micelles barely maintain their spherical shape (Figures 4 and S4). Furthermore, the observed height of the structures of ∼8 nm is significantly smaller than expected from the independently recorded DLS data (Figure 3b) and cryo-TEM images (Figure 2) and agrees well with the DLS observation of at least partial disassembly of the micelles in diluted or nonselective solutions. The simultaneously recorded phase signal is largely homogeneous across the entire micelle area, pointing to similar mechanical properties of the core and shell regions in the non-cross-linked state (Figure 4). In contrast, both topography and phase images of the CCL sample clearly reveal individual micelles (Figure 4). Remarkably, the CCL micelles largely maintain their size (∼15 nm) and shape even in a dry state. Regarding the morphology, size, and homogeneity, the values for the dried CCL micelles excellently agree with the cryo-TEM and DLS data. The pronounced change of the phase signal predominately in the center of the micelle is evident only for the CCL micelles. This suggests that these brighter regions exclusively manifest the DA-cross-linked core. The observed discrepancy between non-cross-linked and DA-cross-linked micelles indicates an increased rigidity of the CCL micelles, which is likely attributable to a homogeneous distribution of FGE moieties across the core region and a sufficient solubility of the DMTE cross-linker in the core region. AFM investigations of similar CCL micelles validated a predominant occurrence of the DA cross-linking reaction in the central core region due to a high presence of cross-linker and FGE.^39^

Whether these changes relate to the CCL process was further investigated by SERS (see Supporting Note 1.4 for details), although SERS primarily provides information on the core–shell interface. SERS spectra of the non-cross-linked and cross-linked micelles show different peak patterns and signal-to-noise ratios (Figure S6). The latter might be related to the lower rigidity of the non-cross-linked micelles, leading to a partial disassembly of the micelles upon interaction with the SERS substrate. SERS of CCL micelles, which nevertheless contain the unreacted DTME cross-linker, detects marker bands (Table S2) associated with the DTME disulfide bridge [ν(S–S) (∼511 cm^–1^)] and the unreacted furfuryl moiety [ν(C=C) (∼1500 cm^–1^)]. These marker bands can be identified also for the DTME CCL micelles. Additionally, characteristic marker bands associated with the DAP [ν(C=C) (∼1514 cm^–1^) and ν(C=O) (∼1761 cm^–1^)] are clearly present for the cross-linked sample but absent for the non-cross-linked sample. The presence of reacted and unreacted species coincides well with the investigations of CCL micelles, which clearly identify a breakdown of the cross-linking process at the core–shell interface.^39^

### 3.3 Quantitative Determination of the Nanomechanical Properties of DTME-Cross-Linked and TCEP-De-Cross-Linked α-Allyl-PEG_80_-*b*-P(*t*BGE_52_-*co*-FGE_12_) Micelles

**Figure 5:**
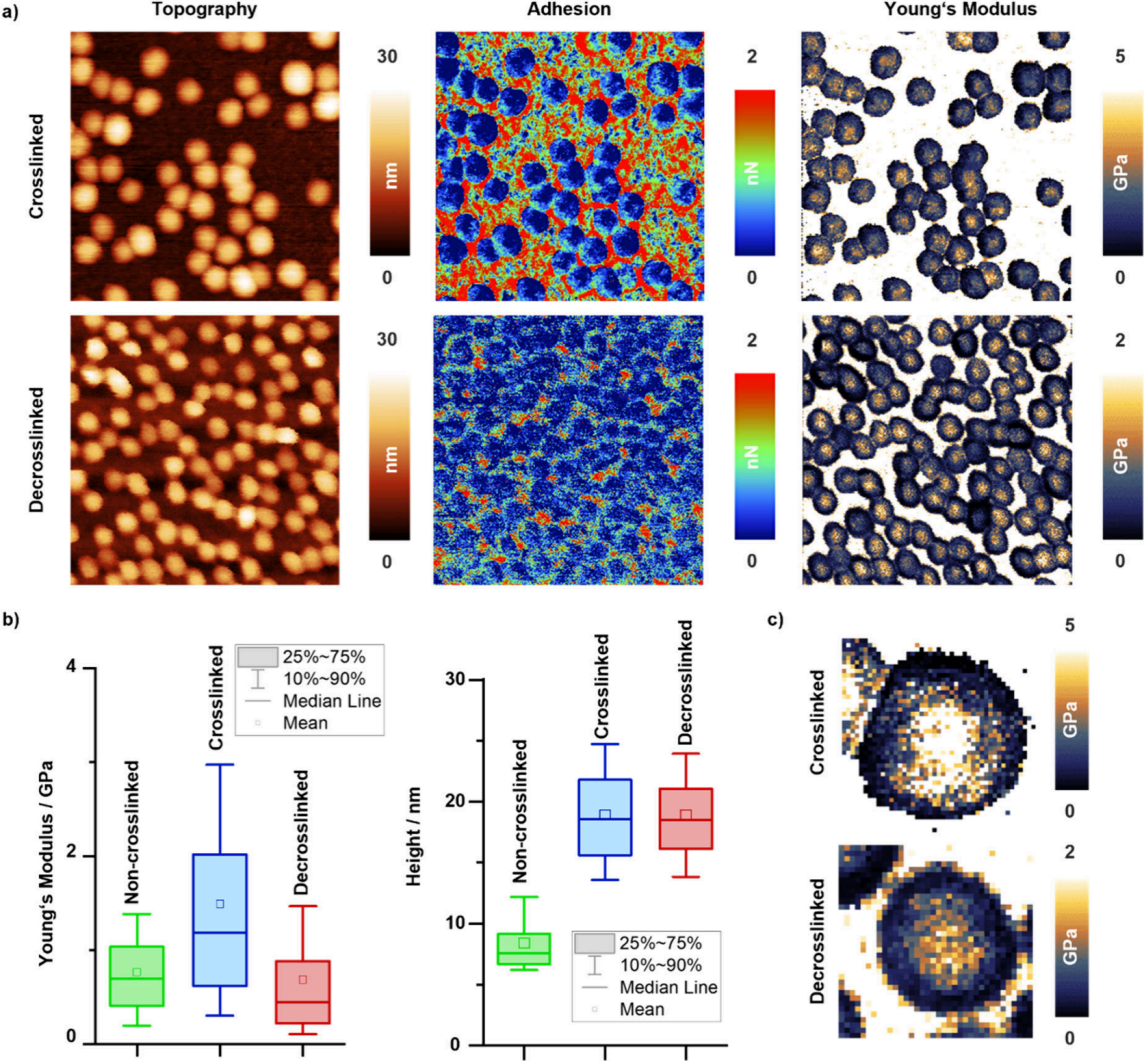
(a) Representative topography, adhesion, and Young’s modulus maps of DTME-cross-linked α-allyl-PEG_80_-*b*-P(*t*BGE_52_-*co*-FGE_12_) micelles (top) and de-cross-linked α-allyl-PEG_80_-*b*-P(*t*BGE_52_-*co*-FGE_12_) micelles (bottom). Image size: 500 nm × 500 nm. (b) Statistical evaluation of the Young’s modulus (left) and height (right) changes for the non-cross-linked (green), DTME-cross-linked (blue), and TCEP-de-cross-linked (red) micelles. (c) High-magnification Young’s modulus maps of cross-linked (top) and de-cross-linked (bottom) micelles; each image is 70 nm × 70 nm. The images illustrate the core–shell structure and the difference in the Young’s modulus caused by de-cross-linking.

While the phase images have effectively delineated the emergence of a distinct core–shell structure, it is essential to acknowledge that this qualitative assessment encompasses various mechanical properties. Consequently, to attain a more granular and quantitative analysis, we shift the capabilities of nanoindentation onto intact micelles in an aqueous environment. This approach permits a detailed examination of the mechanical intricacies responsible for the observed morphological transformations. Figure 5a shows topography, adhesion, and Young’s modulus maps of DTME-cross-linked and TECP-de-cross-linked micelles measured in H_2_O, revealing a homogeneous distribution of spherical micelles. The mean micellar height remains at approximately 19 nm after de-cross-linking. This behavior is consistent with observations made during DLS measurements of non-cross-linked, cross-linked, and de-cross-linked micelles (Figure 3b). Compared to the dried micelles (Figure 4), both samples show a larger average height, demonstrating the importance for investigations in liquid environments to avoid drying artifacts. The adhesion maps representing largely the soft PEG shell exhibit a homogeneous distribution of the adhesion across the entire micelle and no significant changes after de-cross-linking (∼1 nN), in accordance with the assumption that the de-cross-linking process only marginally affects the structure and integrity of the PEG shell.

While the adhesion maps exclusively provided insights in the PEG-shell properties, this is not necessarily the case for determination of the elastic properties. The solvated PEG shell in an aqueous environment shows largely liquid-like behavior. Hence, PEG chains are highly flexible, dynamic, and swell in an aqueous environment fostering fluid-like properties, including a high chain mobility and constantly dynamic rearrangement of the PEG chains. Therefore, the solvated PEG shell may exhibit low compressibility. This is consistent with observations from complementary liquid-phase tip-enhanced Raman investigations, which surprisingly showed that information is gathered from the interfacial PEO-glycidyl ether region in spite of its low depth information capabilities (<2 nm). Depending on the molecular weight, geometry, crystallinity and solvent interaction the resulting Young’s modulus of PEO is usually in a range of 100 kPa to several MPa. However, a selective measurement of the elastic properties of the hydrated PEG shell by means of an AFM nanoindentation approach is hardly accomplishable because the measured forces are inevitably influenced by the stiffness of the supporting material, i.e., here the glycidyl ether core and the glass substrate. Although recently several bottom effect correction (BEC) models for elimination of the substrate-induced BEA have been developed, the large Young’s modulus differences between the soft hydrated PEG shell and the semifluidic glycidyl ether core of the composite material prevent their differentiation. Hence, utilizing the common Hertz contact model, the resulting Young’s modulus values are largely governed by the elastic properties of the micellar core and, thus, provide access to the relative mechanical changes imposed by launched cross-linking and de-cross-linking processes in the confined core region. The central aim of this work is to trace the core-related changes in the mechanical properties, because those specific for the shell will not be affected by the applied chemical modifications. Therefore, focusing on the apparent Young’s modulus values in this study will be sufficient to extract relative changes of the elastic modulus. However, quantification of the real Young’s modulus values would necessitate BEC.^34,35,50^

The Young’s modulus maps (Figure 5a) of the cross-linked and de-cross-linked micelles show a significant decrease of the Young’s modulus after de-cross-linking with average values of 1.18 and 0.68 GPa, respectively. Maps at higher magnification reveal a distribution that resembles the expected core–shell morphology of these spherical micelles (Figure 5c). This core–shell morphology is also reflected in representative line profiles (Figure S4) as well.

Figure 5b shows a quantitative assessment of the stiffness (left) and height (right) changes induced by the cross-linking and de-cross-linking processes in the core region and compares these properties to a non-cross-linked micelle sample containing the unreacted DTME cross-linker, i.e., prior to initiation of the cross-linking process (Figure S4 and Supporting Note 2.3). Despite substantial height variations, the average Young’s modulus of the investigated structures was determined to be ∼0.76 GPa. To a first approximation, this value can be considered as a reference of the Young’s modulus of the non-cross-linked micelles. The nonuniform height and Young’s modulus distributions of the non-cross-linked micelles suggest that morphological changes, likely due to concentrations below the CMC, may lead to disassembly and rearrangement. Conversely, cross-linked and de-cross-linked micelles exhibited uniform size distributions. Box plots of the Young’s modulus of the micellar core regions for each sample, evaluated by considering >150 individual intact micelles across several sample regions for each sample, demonstrate clear changes for this transition. The core region of non-cross-linked micelles exhibits a mean Young’s modulus of 0.69 GPa, whereas the values extend over a range from 0.19 to 1.38 GPa. These values are in good agreement with reported Young’s modulus values for similar PEO_330_-*b*-PFGE_20_ films, which yield 0.46 and 0.91 GPa in the non-cross-linked and cross-linked states.^51^ PFGE has a higher stiffness after cross-linking, reaching 5.13 GPa for a block copolymer with a high number of FGE units. Here the relative number of cross-linkable FGE units is lower, still leading to a degree of cross-linking of more than 75%. Consequently, subtle differences in the stiffness of the PEO corona and the glycidyl ether core are to be expected. Consequently, our investigations measure primarily the nanomechanical properties of the core region and not the PEO shell due to the unavoidable bottom effect.^34,35,47^ After DTME cross-linking of the core, the mean Young’s modulus increases to 1.18 GPa, and the values distribute over a range of 0.30–2.97 GPa. Subsequent de-cross-linking “softens” the core again to values close to the initial state (non-cross-linked). Considering BEAs,^34,35,47^ all Young’s modulus values reported above are therefore apparent and not exact real values. However, considering that the substrate stiffness is not changing for the corresponding samples and that the only minor height difference for cross-linked and de-cross-linked micelles is observed, to a first approximation, similar shifts of the Young’s modulus values to higher values than the real Young’s modulus can be expected. Aimed at revealing the relative changes induced by the cross-linking and de-cross-linking effect, a comparison of the apparent Young’s modulus values is therefore sufficient. Taking into account an overall micellar size of 30 nm (DLS), the herein-reported indentation depth on cross-linked micelles is ∼1 nm and well within the limit of 10% (Figure S6). Most importantly, a strong bottom effect was not observed in a comparison of the small and large features within the non-cross-linked and de-cross-linked samples, pointing toward rather small substrate-induced artifacts. Consequently, the decrease in the Young’s modulus from the cross-linked micelles implies a high efficiency of the TECP de-cross-linking process, which enables reversal of the core-cross-linking to a large extent. The Young’s modulus distributions of the cross-linked and de-cross-linked samples show a higher asymmetry than the sample prior to cross-linking, reflecting most likely the presence of cross-linked and de-cross-linked states due to, i.e., regions of higher and lower (de-)cross-linking efficiency. Notably, the similar Young’s modulus values of non-cross-linked and de-cross-linked micelles might be slightly affected by a more pronounced bottom effect in the case of the non-cross-linked micelles due to larger height deviations and a lack of core-cross-linking, thus leading presumably to a larger uncertainty of their stiffness.

Largely equal height distributions associated with cross-linked and de-cross-linked micelles point to excellent mechanical stability in media diluted below the CMC, in contrast to non-cross-linked micelles, which show a decline of the mean height by a factor of 2. In this case, the entire loss of morphological integrity across all micelles, i.e., none of the micelles reach the height values found in other samples (cross-linked and de-cross-linked), most likely relates to dilution below the CMC. Although the medium in all cases was changed to deionized water after immobilization of the micelles, breaking the disulfide bond by TECP maintains the formed DAP and thus apparently leads to different CMCs for the de-cross-linked and non-cross-linked micelles. Further investigations addressing the chemical composition across the core, shell, and particularly the core–shell interfacial region are required to gain further insights into the solubility of DTME and TECP, the formation of the DAP and the disulfide bond splitting. Multiparametric and multimodal approaches pairing complementary information,^52,53^ e.g., chemical composition and nanomechanical properties, can foster a more detailed understanding of the augmented stability of the de-cross-linked micelles in aqueous environments in the future.

## 4 Conclusions

Cross-linking emerges as a promising strategy for developing advanced DDSs with higher mechanical stability and, potentially, higher degrees of temporal and spatial release control. The presented investigations emphasize the pivotal role played by the selected cross-linking strategy in protecting micelles from unexpected dissociation or drug release. A critical aspect in the construction of self-assembled systems for drug delivery lies in their ability to maintain stability and to resist dissociation. Utilizing AFM and nanoindentation provides the opportunity to comprehensively access the properties of intact DA CCL micelles and monitor their behavior during de-cross-linking, particularly in their native environment. To study this, the micelles were immobilized on a thiol-modified surface via the allyl end groups of the utilized block copolymer and monitored by using AFM nanoindentation in an aqueous environment.

According to the AFM, AFM nanoindentation, SERS, and ^1^H HR-MAS NMR results, DTME demonstrates successful cross-linking of the core via a DA reaction. Moreover, nanoindentation reveals that the reducing agent TCEP initiates the de-cross-linking process efficiently, leading only to minor size and morphology changes, while the mechanical properties of micelles are strongly altered due to effective cleaving of the disulfide bridges introduced by these cross-linkers. It is likely that these micelles would be more permeable to both the surrounding buffer and any potential payload after de-cross-linking, leading to accelerated release. Notably, DTME-cross-linked micelles exhibit de-cross-linking within a day. The findings suggest that disulfide-bridged bismaleimides present a viable option for reversible crosslinking of block copolymer micelles. The susceptibility of non-cross-linked micelles to disassemble due to dilution below the CMC emphasizes the pivotal role of cross-linking in extending the application potential of micelles as drug carriers. The use of DA reactions for cross-linking surely represents a limitation for the types of drugs that can be encapsulated because these must be stable against the reaction conditions in aqueous environments and hydrophobic and can be neither dienes nor dienophiles, so as not to act as a competing reactant. Further research aims at both compatible drugs as well as alternative cross-linking procedures. Nevertheless, the herein-presented data enable us to study block copolymer micelles, which can be regarded as a model system.

Importantly, this investigation was conducted in a liquid environment, enabling a profound understanding by tracking changes in intact micelles, with potential benefits extending to encapsulation processes, and avoiding drying artifacts. Methods that can be used alongside AFM and AFM nanoindentation, such as TERS or Nano-IR spectroscopy, are also promising, potentially enabling the tracking of chemical changes in parallel with the monitoring of changes in the mechanical properties during de-cross-linking or tracking of the release of encapsulated payloads.

## Supporting Information Available

Synthesis of α-allyl-ω-hydroxy-PEG (Supporting Note 1.1), schematic representation of the PEO synthesis initiated with DPMK (Scheme 1), preparation of α-allyl-PEG-*b*-P(*t*BGE-*co*-FGE) (Supporting Note 1.2), determination of the degree of cross-linking (Supporting Note 2.1), amount of unreacted furfuryl and maleimide units in cross-linked micelles (Table S1), DLS examination of non-cross-linked, cross-linked, and de-cross-linked micelles in water and methanol (Supporting Note 2.2), DLS-CONTIN plots of allyl-PEG_80_-*b*-P(*t*BGE_54_-*b*-FGE_14_) micelles with 1 equiv of DTME before and after cross-linking (Figure S1), DLS-CONTIN plots of allyl-PEG_80_-*b*-P(*t*BGE_54_-*b*-FGE_14_) micelles with 1 equiv of DTME after incubation with varying amounts of TCEP and dialysis against water (Figure S2), DLS-CONTIN plots of allyl-PEG_80_-*b*-P(*t*BGE_54_-*b*-FGE_14_) micelles with 1 equiv of DTME after incubation with varying amounts of TCEP and dialysis against water, followed by dialysis against methanol (Figure S3), AFM nanoindentation of intact, immobilized micelles in aqueous solutions (Supporting Note 2.3), height and Young’s modulus variations of non-cross-linked, CCL, and de-cross-linked micelles determined from selected line profiles of the corresponding AFM images (Figure S4), representative force–distance curves of intact non-cross-linked (left), cross-linked (middle), and de-cross-linked (right) micelles in an aqueous environment (Figure S5), SERS of non-cross-linked and cross-linked micelles in H_2_O (Supporting Note 2.4), SERS spectra of non-cross-linked and CCL allyl-PEG_80_-*b*-P(*t*BGE_54_-*b*-FGE_14_) micelle solutions (Figure S6), and characteristic Raman marker bands of cross-linked and non-cross-linked micelles (Table S2) (PDF)
