# Supplementary material for: Nanoscale Investigation of Elasticity Changes and Augmented Rigidity of Block Copolymer Micelles Induced by Reversible Core-Cross-Linking
Source: ACS Appl Mater Interfaces. 2025 Apr 23;17(18):27557–67. doi: 10.1021/acsami.5c04826 (PMC12067378; doi:10.1021/acsami.5c04826)
Supplement: Supplementary file 1 — am5c04826_si_001.pdf [file am5c04826_si_001.pdf]

Supporting Information

Nanoscale Investigation of Elasticity Changes and Augmented Rigidity of Block Copolymer Micelles Induced by Reversible Core-Crosslinking

Xinyue Wang[1,2], Andreas Stihl[3,4], Christiane Hoeppener[1,2,*], Jürgen Vitz[3], Felix H. Schacher[3,4*] and Volker Deckert[1,2,4]

[1]Institute of Physical Chemistry and Abbe Center of Photonics, Friedrich-Schiller University, D-07743 Jena, Germany

[2]Leibniz Institute of Photonic Technology, D-07745 Jena, Germany

[3]Institute of Organic Chemistry and Macromolecular Chemistry, Friedrich-Schiller University, D-07743 Jena, Germany.

[4]Jena Center for Soft Matter (JCSM), Friedrich Schiller University Jena, Philosophenweg 7, D-07743 Jena (Germany)

*Corresponding authors. E-mail: christiane.hoeppener@uni-jena.de;

felix.schacher@uni-jena.de

1. Materials and Methods

Reagents and solvents were commercial products purchased from Sigma Aldrich or Linde. Prior to use, diphenylmethane (DPM) was stirred over calcium hydride and then distilled under vacuum. HPLC quality THF was obtained from a solvent purification system (SPS) and further dried by heating to reflux over freshly prepared sodium-benzophenone until a deep blue color appeared and distilled. The purified reactants were stored in Schlenk flasks in a glove box, flushed with argon and used within a few days. Ethylene oxide (EO) was purified by stirring over sodium metal and subsequent distillation. All solvents utilized for the MALDI-TOF-MS measurements were LC-MS grade, purchased from Sigma Aldrich, and used as received.

Potassium hydride was purchased as a 25-35w% suspension in mineral oil from Acros Organics, washed using dry cyclohexane under an argon atmosphere, and dried under vacuum. Dibenzo 18-crown-6 (DBCE, 98%) and dithiobismaleimidoethane (DTME, >90%) were purchased from Sigma Aldrich and used as received. Tetrahydrofurane (THF) was purchased from VWR and distilled under reduced pressure. For use in AROP, it was further dried by refluxing over sodium/benzophenone, distillation under argon and storage over 4 Å-molecular sieve in an argon-filled glovebox. All polymerizations were carried out using oven-dried glassware in an argon-filled glovebox. $CDCl_3$ was purchased from Deutero and used as received. Tris(2-carboxyethyl)phosphine-hydrochloride (TCEP) was purchased as a 0.5 M solution (pH=7, buffered with ammonium hydroxide) and as a solid from Sigma Aldrich.

1.1. Synthesis of α-allyl-ω-hydroxy-PEG

The used glassware was cleaned and dried in an oven at 105 °C before use. The glassware transferred to the glove box was exposed to vacuum, followed by flushing with nitrogen. This

step was repeated three times to remove possible impurities. The polymerization was performed in a small scale PicoClave glass autoclave (250 mL) (Büchi AG, Uster, Switzerland). Before use, the autoclave was cleaned with water and acetone, subsequently dried under vacuum for 24 hours and flushed with argon. The system is equipped with a stainless steel cover, fast action closure, pressure gauge, different valves, a rupture disc, and a polycarbonate shield for safety. The EO is stored in a glass and stainless made burette rated up to 12 bar, equipped with a heating/cooling jacket and connected to the autoclave via a mass flow controller. In addition, the autoclave system and the burette are equipped with temperature and pressure sensors. For automation, a Siemens Simatic S7-1200 was used (Siemens AG, Munich, Germany). To control the ethylene oxide addition, a Bronkhorst mini CORI-FLOW device (M12V14I-PGD-22-K-S, Bronkhorst High-Tech B.V., Ruurlo, Netherlands) was used, allowing flow rates between 0.1 and 200 g/h with an accuracy of ±0.2%. For heating/cooling, a Huber Unistat 390w chiller was used, offering a working temperature range between –90 °C up to 200 °C (Huber Kältemaschinenbau GmbH, Offenburg, Germany).

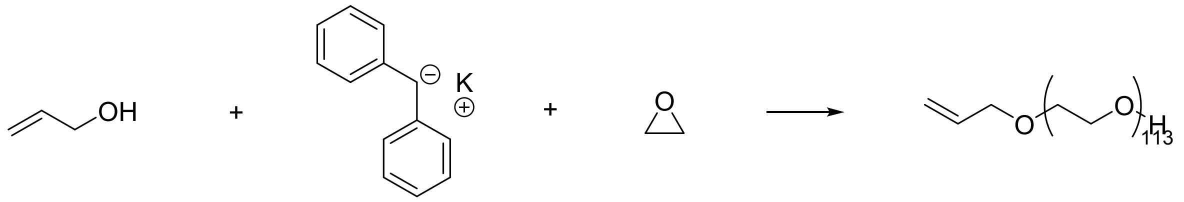

Scheme S1. Schematic representation of the PEO synthesis initiated with diphenylmethyl potassium (DPMK).

The synthesis of the polymers was performed in the aforementioned PicoClave autoclave. The general route is shown in Scheme 1. In the glove box, 2-(allyloxy)ethan-1-ol (174 mg) and

dry THF (100 ml) was introduced into a 250 mL glass bottle. Afterwards, the solution was transferred to the Büchi reactor, and the allyl alcohol was titrated with potassium diphenylmethanide (4.21 ml, 3.00 mmol) at 10 °C until the light yellow color remained. Then, oxirane (15 g, 17 ml, 340 mmol) was added controlled via the mass flow controller. The reactor temperature was increased from 10 °C to 45 °C within 60 min while stirring. After 24 h, the pressure remained constant at 0.2 bar and the EO atmosphere was released in an iso-propanol/KOH solution. In the reactor no formation of gas was visible anymore, and hence the reaction seemed to be complete.[1] After evaporation of the solvent mixture, the crude product was redissolved in pure THF and precipitated twice in cold ether, filtered, and dried under vacuum to obtain the product a white powder.

^1H NMR (300 MHz, CD_2Cl_2): δ (ppm) = 5.88-5.82 (allyl-H, 1H), 5.29-5.17 (allyl-H, 2H), 4.00-3.98 (PEO backbone), 3.86-3.39 (PEO backbone), ^1H NMR: Mn = 5000 g/mol; SEC: M_n = 3200 g/mol, Mw = 3400 g/mol, Đ = 1.08, MALDI-TOF-MS: Mn = 3600 g/mol. For further calculations, M_n = 3.5 kg/mol was assumed.

1.2. Preparation of *α*-allyl-PEG-*b*-P(*t*BGE-*co*-FGE)

α-allyl-*ω*-hydroxy-PEG (M_n=3.5 kg/mol, 150 mg, 43 µmol, 1eq.) is dissolved in THF (1.5 mL) in a microwave vial. Potassium hydride (1.4 mg, 36 µmol, 0.84 eq.) and Dibenzo-18-crown-6 (13.0 mg, 36 µmol, 0.84 eq.) are added, the vial is sealed with a septum cap and the mixture is heated to 70 °C for 75 minutes. Afterwards, a solution of tert-butyl glycidyl ether (312 mg, 340 µL, 2.4 mmol, 56 eq.) and furfuryl glycidyl ether (92.4 mg, 83 µL, 0.6 mmol, 14 eq.) in THF (0.5 mL) is added through the septum using a syringe. The mixture is stirred at 70 °C for 40 h. The reaction is quenched by the addition of excess methanol (1 mL). The

polymer is purified *via* dialysis (MWCO=1 kDa, methanol:THF 1:1 v:v) and dried under vacuum. Two batches of the polymer were prepared. Batch 1 was used for AFM, SERS, (cryo-)TEM and HR-MAS-NMR investigations, batch 2 was used for DLS investigations.

Batch 1:

^{1}H NMR (300 MHz, $CDCl_3$): δ = 7.37 (s, Ar-H), 6.30 (s, Ar-H), 4.45 (s, Ar-CH_2), 3.66-3.35 (m, Backbone), 1.16 (s, *t*-Bu) ppm, resulting in: allyl-PEG_{80}-*b*-P(*t*BGE_{52}-*co*-FGE_{12})

SEC (THF, PEG calibration): M_n= 8.3 kg/mol, PDI= 1.4

Batch 2:

^{1}H NMR (300 MHz, $CDCl_3$): δ = 7.37 (s, Ar-H), 6.30 (s, Ar-H), 4.45 (s, Ar-CH_2), 3.66-3.35 (m, Backbone), 1.16 (s, *t*-Bu) ppm, resulting in: allyl-PEG_{80}-*b*-P(*t*BGE_{54}-*co*-FGE_{14})

SEC (THF, PEG calibration): M_n= 8.8 kg/mol, PDI= 1.22

2. Supporting Results and Discussion

2.1. Determination of the degree of crosslinking

Degree of crosslinking was calculated as described in Ref.[2]. The molar ratios of unreacted furfuryl and maleimide with regards to the Diels-Alder product were determined using ^{1}H-HR-MAS-NMR, as seen in Figure **3a**. The upper limit was set assuming all unreacted maleimide units stem from encapsulated DTME, while the lower limit was set assuming all such units stem from monoreacted DTME. The results are summarized in Table **S1**.

Table S1. Amount of unreacted furfuryl and maleimide units in crosslinked micelles of α-allyl-PEG$_{80}$-*b*-P(tBGE$_{52}$-*co*-FGE$_{12}$) as a percentage of initially present FGE-units, as determined using ^{1}H-HR-MAS-NMR, with different amounts of DTME as equivalents of initially present FGE units.

Amount of Crosslinker	Unreacted Furfuryl units	Unreacted Maleimide units	Degree of Crosslinking
0.0 eq.	100%	-	-
0.65 eq.	50%	14%	36-50%
1.00 eq.	12%	13%	75-88%

2.2. DLS Examination of non-crosslinked, crosslinked and decrosslinked micelles in water and methanol

DLS examination of the micelle solutions incubated with increasing amounts of TCEP show a slight increase in the micellar size, probably caused by initiation of core decrosslinking and an accompanying mechanical destabilization. Solvent exchange to methanol lead already to slight swelling of the micelles in the core-crosslinked state (see control samples in Figure **3**), and hence, to overall larger micellar size changes. Despite swelling neither in H_2O nor in MeOH an entire disassembly of the micelles is observed, indicating a substantial mechanical integrity of the decrosslinked micelles. This is consistent with the increased micelle stability after crosslinking and decrosslinking observed in our AFM studies. The number-weighted CONTIN-plots obtained for the micelle solutions are shown in Figures **S1-S3**.

Although the mean hydrodynamic radii indicated that decrosslinked micelles are not susceptible to a high degree of disassembly, a closer inspection of the DLS CONTIN-plots recorded in the non-selective solvent (methanol) shows a greater heterogeneity in these results (see Figure **S3**). Multimodal distributions with a considerable population in a range around $r_H \leq 10$ nm, i.e., significantly smaller hydrodynamic radii than what was observed for intact micelles, being detected in some instances. Particles in this size range may be attributed to fragments of micelles or free polymer. In contrast DLS CONTIN-plots of decrosslinked micelle solutions in water always yielded r_H values above 10 nm (see Figure **S2**). Due to Rayleigh scattering intensity scaling with r^6, one possible explanation for this is that a population of fragments of decrosslinked micelles exists in these methanol solutions, which is only detected in some instances due to the presence of still intact micelles whose larger size allows them to overwhelm the light scattered by the fragments.

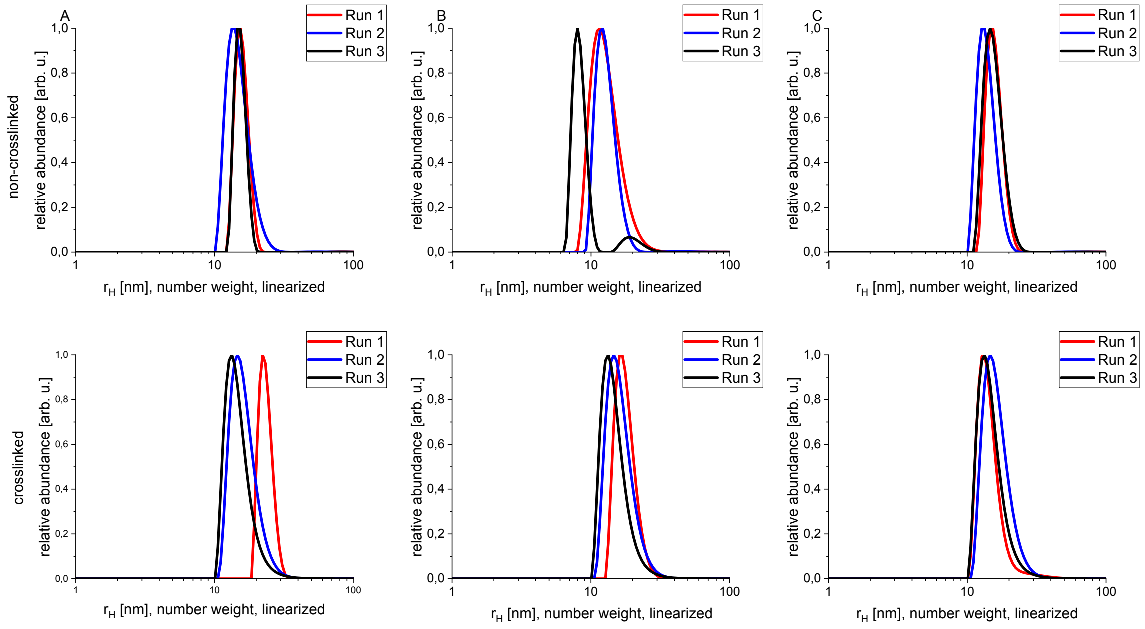

Figure S1. DLS-CONTIN-plots obtained for micelles of allyl-PEG_{80}-*b*-P(*t*BGE_{54}-*b*-FGE_{14}) w. 1eq. of DTME before and after crosslinking. Experiment performed at least in triplicate, results for individual samples shown in columns A, B and C.

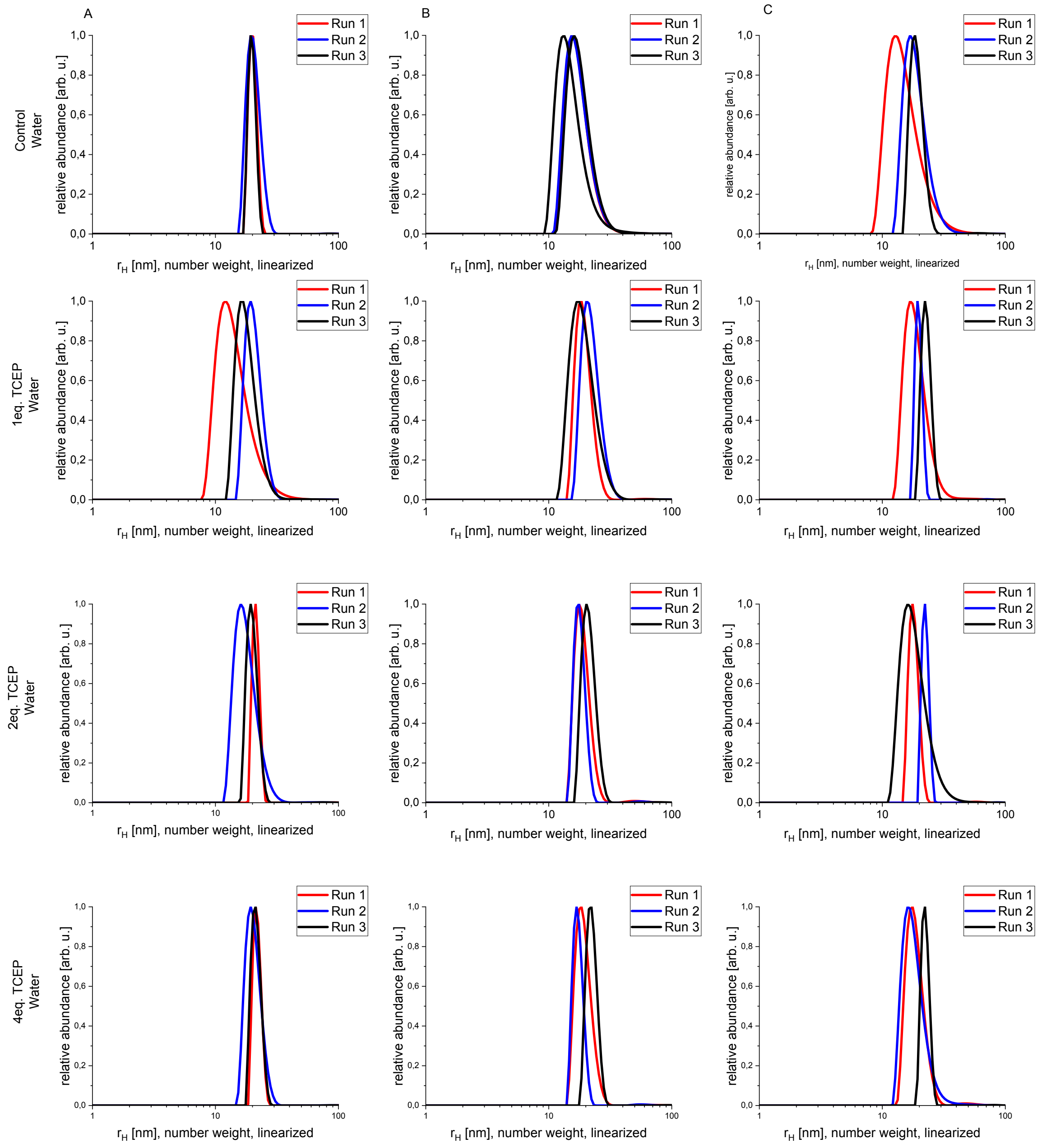

Figure S2. DLS-CONTIN-plots obtained for micelles of allyl-PEG$_{80}$-*b*-P(*t*BGE$_{54}$-*b*-FGE$_{14}$) w. 1eq. of DTME after incubation with varying amounts of TCEP and dialysis against water. Experiment performed in triplicate, results for individual samples shown in columns A, B and C.

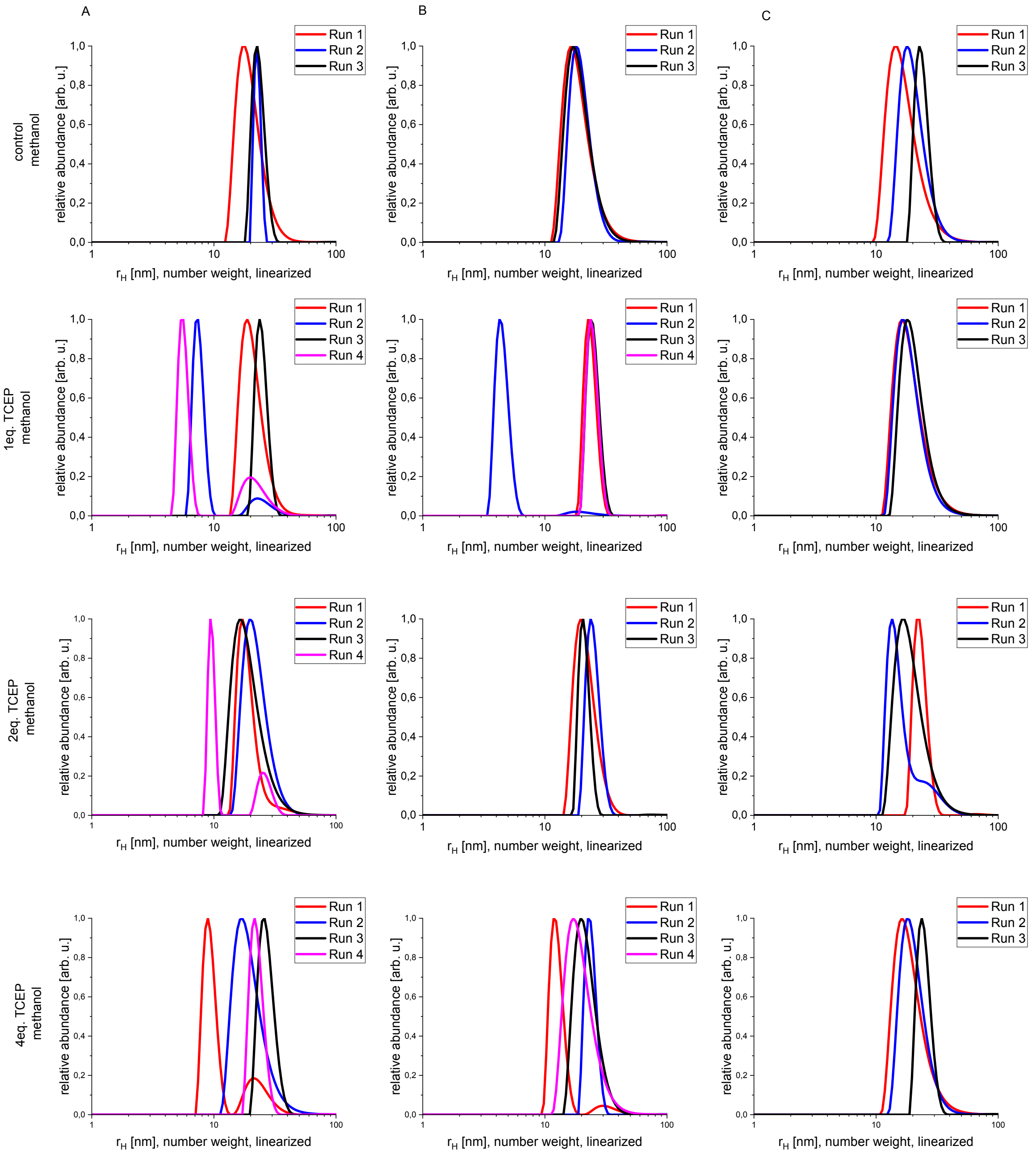

Figure S3. DLS-CONTIN-plots obtained for micelles of allyl-PEG$_{80}$-*b*-P(*t*BGE$_{54}$-*b*-FGE$_{14}$) w. 1eq. of DTME after incubation with varying amounts of TCEP and dialysis against water, followed by dialysis against methanol. Experiment performed in triplicate, results for individual samples shown in columns A, B and C.

2.3. AFM Nanoindentation of intact, immobilized Micelles in aqueous solutions

In the topography and Young's modulus images of non-crosslinked micelles (Figure **S4**), no discernible core-shell structure is observed, and both the height and size exhibit non-uniformity. Only a small part of these micelles can still maintain the shape after immobilization as well as during the nanoindentation measurement in H_2O. Due to the strict limitation CMC, several micelles, or at least parts of the copolymer forming bonds with the thiol group, could remain on the functionalized glass. Possible rearrangement and corresponding CMCs could also occur with the polymer from residues of broken micelles. Despite substantial variations in the line profiles (1–3) corresponding to representative micelles with differing heights, ranging from 1 to 10 nm, they consistently exhibit a Young's Modulus of approximately 0.3 GPa – 0.5 GPa. This value can be considered indicative of the Young's Modulus for non-crosslinked micelles. In contrast, the profiles of representative crosslinked and decrosslinked micelles showed similar heights, while the Young's modulus decreased from 1 GPa - 2 GPa to less than 1 GPa after the decrosslinking process. Additionally, all Young's modulus images and profile lines of crosslinked and decrosslinked micelles showed a distinct core region.

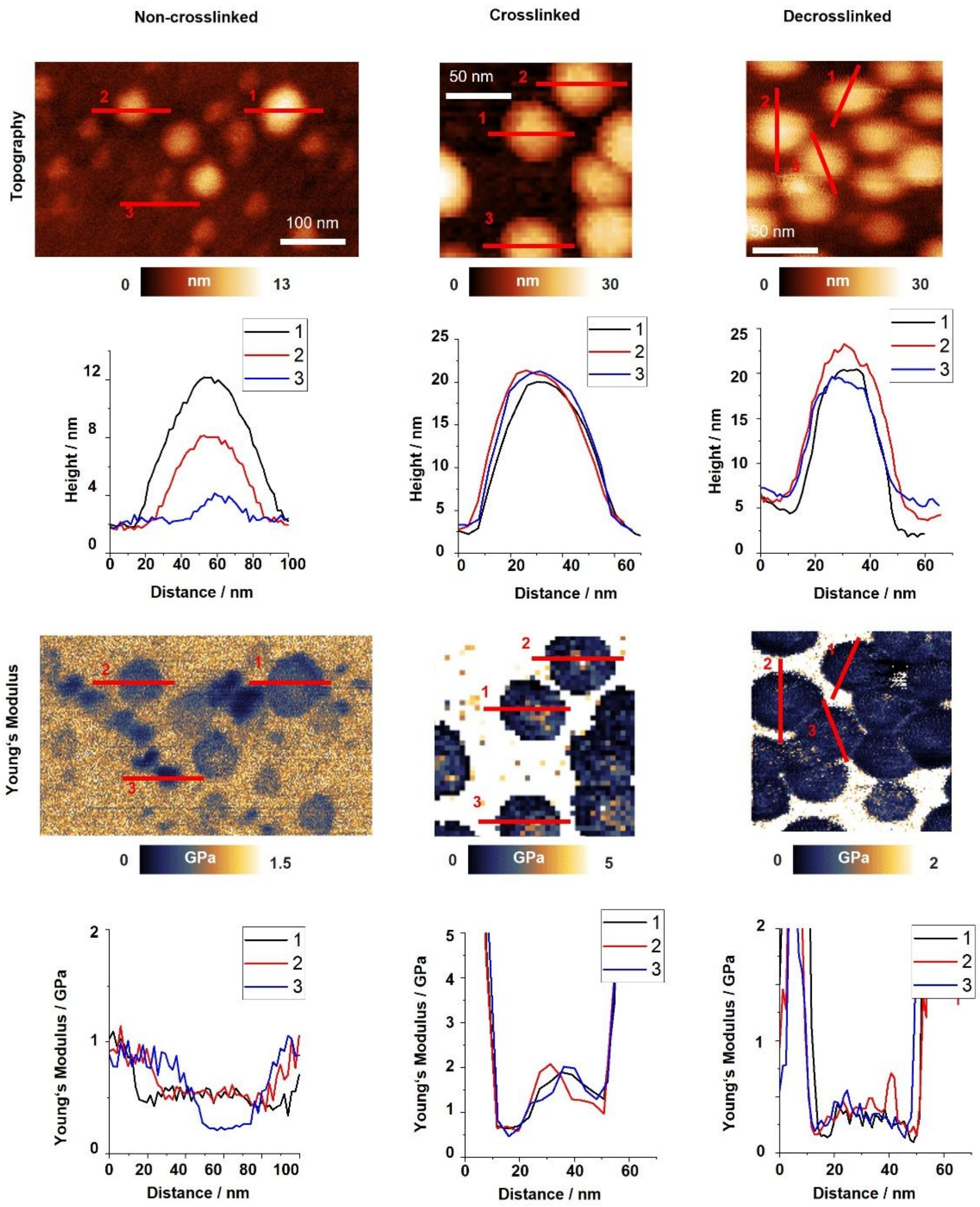

Figure S4. Nanoindentation investigations of intact non-crosslinked, crosslinked and decrosslinked micelles in aqueous environment. Top- Topography images and three extracted line profiles showing distinct height variations. Bottom- Young's modulus maps of the same region evaluated from simultaneously recorded slope (force constant) images. The lineprofiles were extracted at the indicated positions.

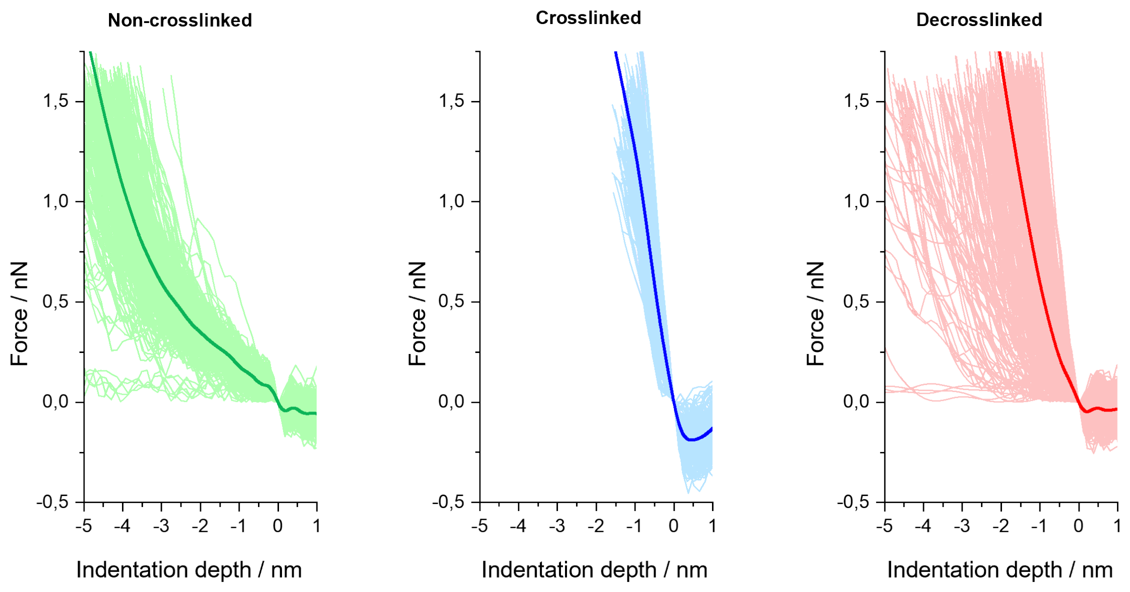

Figure S5. Representative force-distance curves of intact non-crosslinked (left), crosslinked (middle) and decrosslinked (right) micelles in aqueous environment.

Figure S5 presented 300 FD-curves recorded across an area of 20 x 20 nm^2 at the center of three different micelles of non-crosslinked, crosslinked and decrosslinked samples, thus representing the central core region and the interfacial region. By setting a constant applied force of ~1.5 nN, an indentation depth of 1 nm on crosslinked micelles was observed. The same applied force led to varying indentation depths, resulting most probably from variations in their elasticity induced by the applied chemical reactions. Deeper indentation on "softer" positions, is especially observed in non-crosslinked and decrosslinked micelles. Particularly FD curves with indentation depth of 3-4 nm can be assigned to the interfacial regions or core regions with a high degree of decrosslinking. The relatively large spread can be explained by edge effects related to the investigation of intact micelles and might be partly affected also by the bottom-stiffness. Therefore, a mask based on height was applied for further statistic analysis of the Young's Modulus maps shown in Figure 5b to reduce these effects.

2.4. Surface enhanced Raman spectroscopy of micelle solutions and band assignment

Micelle solutions were dropped on Ag-SERS substrates fabricated to the procedure described in Ref. [3]. All measurements were carried out without drying steps in an aqueous solution. The sample was excited with a linearly polarized laser beam with an excitation wavelength of 532 nm. The excitation power at the sample was ~ 187 µW. Each spectrum was acquired in 1 s and accumulated for 20 times. Spectra were intensity normalized into the range [0,1]. No smoothing or background correction was applied (Figure **S6**).

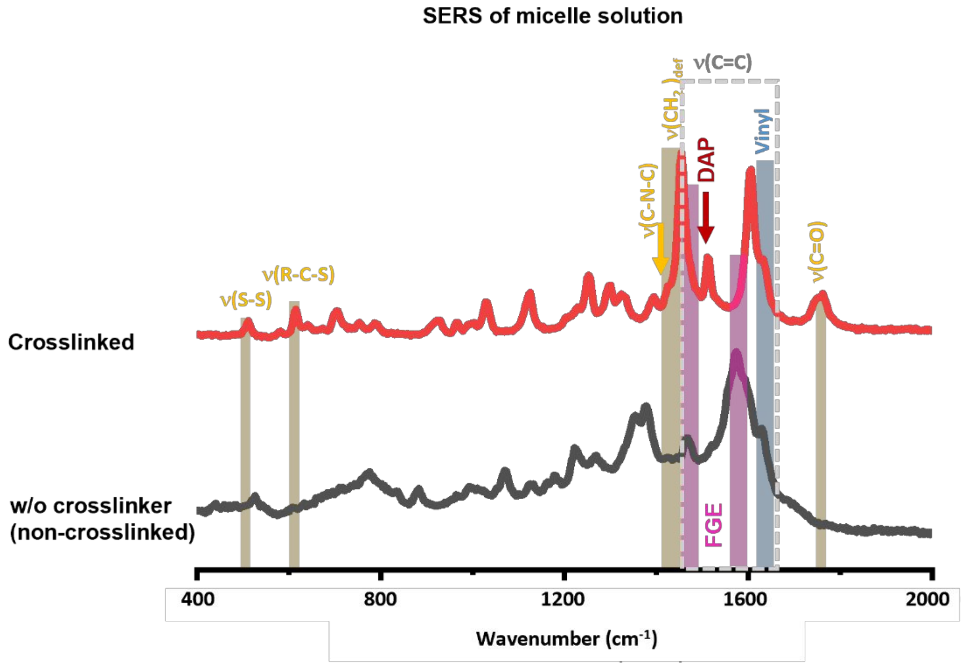

Figure S6. SERS investigation of the crosslinked (red, top) and non-crosslinked (grey, bottom) micelle solutions. Several marker bands are detected which can be uniquely assigned to the DTME crosslinker, the formed DAP product and unreacted FGE moieties of the hydrophobic glycidyl ether block of the block copolymer (see Table **S2**).

Table S2. Representative Raman marker bands of crosslinked micelles and non-crosslinked micelles without crosslinker (wavenumber in cm^{-1}). Vibrational mode assignment according to Ref. [4-9]

	Band	crosslinked	non-crosslinked (w/o crosslinker)
DTME/DAP	$\nu(S-S)$	511	
	$\nu(C-S)$	615	
		641	
		673	
	$\nu(CH_2)$	1252	
	$\nu(C-N-C)$	1427	
	$\nu(CH_2)$	1454	
DAP	$\nu(C=C)$	1514	
FGE	$\nu(C=C)$		1467
	$\nu(C=C)$		1573
Vinyl	$\nu(C=C)$	1628	1628
DTME/DAP	$\nu(C=O)$	1761	

REFERENCES

(1) Vitz, J.; Majdanski, T. C.; Meier, A.; Lutz, P. J.; Schubert, U. S. Polymerization of ethylene oxide under controlled monomer addition via a mass flow controller for tailor made polyethylene oxides. *Polym. Chem.* **2016,** *7* (24), 4063-4071, DOI: **10.1039/c6py00402d**.
(2) Elter, J. K.; Sentis, G.; Bellstedt, P.; Biehl, P.; Gottschaldt, M.; Schacher, F. H. Core-crosslinked diblock terpolymer micelles – taking a closer look on crosslinking efficiency. *Polym. Chem.* **2018,** *9* (17), 2247-2257, DOI: 10.1039/c8py00126j.
(3) Wang, L.; Womiloju, A. A.; Höppener, C.; Schubert, U. S.; Hoeppener, S. On the stability of microwave-fabricated SERS substrates - chemical and morphological considerations. *Beilstein J. Nanotechnol.* **2021,** *12*, 541-551, DOI: 10.3762/bjnano.12.44.
(4) Scherer, J. R.; Overend, J. Transferability of Urey ‐ Bradley Force Constants. III. The Vinylidene Halides. *J. Chem. Phys.* **1960,** *32* (6), 1720-1733.
(5) Ignatyev, I. S. Scaled ab initio force fields of s-cis and skew conformers of methyl vinyl ether. *J. Mol. Struct.* **1991,** *246* (3-4), 279-287, DOI: 10.1016/0022-2860(91)80134-p.
(6) Höppener, C.; Elter, J. K.; Schacher, F. H.; Deckert, V. Inside Block Copolymer Micelles-Tracing Interfacial Influences on Crosslinking Efficiency in Nanoscale Confined Spaces. *Small* **2023,** *19* (20), e2206451, DOI: 10.1002/smll.202206451.
(7) Bazylewski, P.; Divigalpitiya, R.; Fanchini, G. In situ Raman spectroscopy distinguishes between reversible and irreversible thiol modifications inl-cysteine. *RSC Adv.* **2017,** *7* (5), 2964-2970, DOI: 10.1039/c6ra25879d.
(8) Kuhar, N.; Sil, S.; Umapathy, S. Potential of Raman spectroscopic techniques to study proteins. *Spectrochim. Acta A Mo.l Biomol. Spectrosc.* **2021,** *258*, 119712, DOI: 10.1016/j.saa.2021.119712.
(9) Socrates, G. *Infrared and Raman characteristic group frequencies: tables and charts*, 3rd. edn. ed.; John Wiley & Sons: The University of West London, Middlesex, U.K., 2004.
